# Ten simple rules for successfully supporting first-generation/low-income (FLI) students in STEM

**DOI:** 10.1371/journal.pcbi.1010499

**Published:** 2022-10-06

**Authors:** Courtney Peña, Nidia Ruedas-Gracia, Jennifer R. Cohen, Ngoc Tran, Miranda B. Stratton

**Affiliations:** 1 Biosciences Grant Writing Academy, Stanford University, Stanford, California, United States of America; 2 College of Education, University of Illinois at Urbana-Champaign, Champaign, Illinois, United States of America; 3 Office of the Vice Provost for Undergraduate Education, Stanford University, Stanford, California, United States of America; 4 Department of Education, University of California, Los Angeles, Los Angeles, California, United States of America; 5 School of Medicine Human Resource Group, Stanford University, Stanford, California, United States of America; Carnegie Mellon University, UNITED STATES

## Introduction

Around 30% of undergraduate college and university students in the United States fall under the category of first-generation and/or low-income (FLI) [1]. Within science, technology, engineering, and mathematics (STEM), the number of FLI students is closer to 20% [2], indicating a disparity in support and accessibility for FLI students in STEM.

FLI students are an important part of the American higher educational landscape yet much of the rhetoric and research on FLI college students is centered on deficit-based frameworks [3] that focus on what they lack, rather than leveraging their strengths. FLI students enrich educational spaces—especially in STEM environments—and should be met with proper support so that they may thrive in their programs. Research shows that FLI students offer robust life experiences and are innovative, collaborative, community-oriented, and creative [3–8]. Educators must be ready to support them through asset-based approaches to improve FLI STEM student retention.

This paper offers 10 simple rules for successfully supporting FLI STEM undergraduate students. The strategies below are informed by research as well as the authors’ experiences as education researchers, practitioners, and former FLI students. It will be most helpful for mentors, advisors, professors, or anyone in a supportive role for FLI undergraduate students in STEM fields.

### Rule 1: Get to know the FLI community on campus

FLI students exist on every campus, yet because this population is centered around socioeconomic status, a student’s FLI identity might not always be apparent or visible, making it difficult to know when to differentiate support. A great first step in supporting FLI students is to get to know what exists and build from there [9]. For example, many campuses have university-sponsored programming and resources, as well as student-led organizations created specifically to support FLI students. You can begin by searching your institution’s website for FLI student resources or by asking colleagues. It might take some work to get to know what organizations and resources exist to support FLI students at your institution, but there are many benefits to being engaged with this community.

It’s important to be proactive in engaging with and understanding the unique needs of this community and understand how multiple identities intersect and impact students’ lived experiences (e.g., gender, disability, immigration status, sexuality, race/ethnicity, generational status). When working with individual students, ask for guidance on how they want to be supported. Don’t assume to know the answers. Listen carefully to student responses to questions like “How can I help you reach your education and career goals?” and “What do you need to feel confident in your abilities to excel in your program?” Additionally, you can begin to build trust with FLI students if they see you attending FLI events, supporting FLI programming, or by providing snacks in your lab meetings, classroom, or office. If you have the means, invite students for working lunch meetings and treat them to a meal.

It’s important to understand the availability of resources specific to your institution type. The type of support available varies based on institutions. For example, community colleges will have different resources and funding than a private 4-year institution. However, minority serving institutions are often more connected to the needs of their students including socioeconomic and identity-based needs. FLI student experiences are vastly different based on the resources available to them within institutions they attend. Understanding the resources offered at your institution will be an important step in fostering collaboration with the FLI community on your campus.

### Rule 2: Check in on basic needs

As supporters of FLI students, it’s important to know what kind of experiences they are having when it comes to meeting basic physiological needs such as food and shelter. For example, it is well documented that food insecurity is alarmingly high at US institutions of higher education [9–13] and has a detrimental impact on academic performance, class attendance, and ability to focus [14]. Housing insecurity has also been a major challenge for college students and poses considerable barriers to learning and overall wellbeing [9,15]. As supporters of FLI students, it’s helpful to know what resources are available at your institution. It might be the case that your university sponsors a community pantry or basic needs center (see examples of university-sponsored efforts such as NC State University’s Feed the Pack program [16] or UC Berkeley’s Basic Needs Center [17]; see an example of national nonprofits such as Swipe Out Hunger [18] which has anti-hunger programs across a network of 400 campuses). Familiarizing yourself with resources and checking in with your students on basic needs can provide you with more information on how to best support them and improve learning conditions.

### Rule 3: Embrace differences in cultural norms

Colleges typically espouse cultural norms aligned with middle-class, independent norms, and practices. These norms are often not matched with the cultural norms that FLI students bring with them to college, which are typically working-class, interdependent, and collectivistic norms and practices [19]. Historically, colleges have favored an assimilative approach to supporting FLI STEM students where FLI STEM students feel like they needed to hide their ways of being to “fit in” or “belong” in college [20]. Instead of looking down on cultural differences between FLI STEM students and their peers, supporters can decrease the cultural mismatch felt by FLI STEM students by embracing differences in cultural norms and welcoming working-class, interdependent traditions into the college setting. This could look like embracing collaborative group science or finding ways for students to bring their families and communities into the work they’re doing. For an example, see the members and philosophy section of the Fraser Lab [21]. In this way, FLI STEM students, along with their non-FLI counterparts, will feel their cultural background is valued, respected, and important within STEM.

### Rule 4: Foster a sense of belonging

Generally, a sense of belonging is defined as having a sense of positive relationships with others [22]. When students have a strong sense of belonging, they see improvements in mental health, academic performance, and retention [23–25]. When students lack a sense of belonging, they experience additional challenges with academic performance and retention and are more at risk for stress, anxiety, and depression [26,27]. Because of the cultural differences described in Rule 3, FLI STEM students can experience additional barriers to feeling like they belong. However, sense of belonging is not fixed and can change over time.

Students can develop a sense of belonging to various contexts within the college such as a STEM major, a research lab, or various organizations/centers. This means that there are many avenues within a college context through which supporters of FLI STEM students can foster belonging, be it by creating positive relationships between FLI STEM students and STEM faculty and peers or by maintaining cultural centers (including FLI centers) where both STEM and non-STEM students can build their sense of belonging. It is important to remember that while a sense of belonging is critical for all students, students from minoritized cultural backgrounds experience additional challenges as sense of belonging is tied to social identities [28]. Supporters of FLI STEM students should create opportunities for students from various minoritized groups (e.g., BIPOC, gender non-conforming, immigrant) to feel welcomed, cared for, valued, and respected [28]. Fostering a sense of belonging includes establishing spaces where FLI STEM students can be their authentic selves [29].

### Rule 5: Understand how financial challenges impact learning and engagement

FLI students often navigate unique financial circumstances that impact their experience in higher education. At the individual level, FLI students may have responsibilities within their families or communities to provide emotional support and advocacy, language brokering, financial support, physical care, life advice, and heavy sibling/parent caretaking [30,31]. Some FLI students pursue part-time or full-time employment to meet financial obligations that can disrupt their academic performance, thus taking time away from social acclimation to the campus community [32]. Lack of college affordability and access to financial aid can extend the time to a degree, result in students incurring more debt, and lead to departure from the institution altogether [33,34]. Academic expenses unique to STEM courses (e.g., scientific textbooks, lab equipment, manuals) can deter FLI students from continuing their STEM degrees. Consider offering affordable alternatives such as an open-exchange STEM library or putting a statement about basic needs in your syllabus to show your commitment to supporting FLI student learning [9] (see [9] for more examples of strategies for faculty). Understanding the socioeconomic realities of FLI students will take time and relationship building. Our next rule will offer guidance on how you can begin to build rapport through empathy and trust.

### Rule 6: Demonstrate empathy and compassion to build trust

There are a lot of stigmas attached to low socioeconomic status, making it difficult for FLI students to disclose their struggles to faculty, mentors, and student services professionals. In fact, some may choose not to disclose at all. To break down some of the stigma, educators can proactively apply empathetic and compassionate practices independent of students’ identity disclosure. This can look like learning about your students and their experiences and adjusting policies as needed [35]. For example, offering flexible meeting times or makeup lab sections with your FLI students can help them balance their multiple demands. Whether you are an administrator, faculty member, or advisor, building trust and demonstrating empathy and compassion with your students is vital in understanding and supporting their needs. As Meyers and colleagues state, “Instructors high in teacher empathy do not lower standards; they identify and remove obstacles to learning” [35]. Campus faculty and staff can create an empathetic, compassionate, and FLI-friendly culture by checking in on FLI students periodically (initiated by the faculty/staff and not by the student). Empathy and compassion are hard work [36] but being a trusted source of support for FLI students could make a critical impact on their learning, retention, and wellbeing.

### Rule 7: Openly discuss impostor syndrome and offer holistic mentoring

Imposter syndrome is an individual’s intellectual self-doubt and fear of failure often accompanied by feelings that they have fooled or deceived people into thinking they are more capable than they perceive themselves to be [37]. Nearly everyone experiences some sort of imposter syndrome at some point in their life. FLI and other marginalized students in STEM experience imposter syndrome, as well as other structural challenges that impact their ability to thrive academically, causing them to question their ability to succeed in STEM’s merit-based culture [38]. Openly discussing imposter syndrome also requires conversations about campus culture and climate. This can include how institutional inequity has disproportionally and historically impacted students with marginalized identities, creating ongoing disparities in STEM. When campus leaders and mentors openly discuss structural barriers to success, FLI students can have more opportunities to be their whole selves in STEM.

Providing explicit support for academic learning will build authentic relationships with FLI students. Ask questions that help you learn more about the lived experiences of individual FLI students and where structural challenges are contributing to feeling othered in STEM. For example, questions like, “What has been your experience been like in STEM as a FLI student? What questions do you have about STEM fields and future careers? What do you need to be successful as a FLI STEM student? What internal and/or external challenges are you facing? How can I support you on your STEM journey?” can build a strong mentoring foundation that shows you value your student’s identity as a scientist alongside their FLI identity and other social identities like race/ethnicity, gender, and generational status. When discussing topics like imposter syndrome, academic struggles, and other structural issues in academia, we suggest offering a holistic approach to help your FLI students thrive and succeed in STEM (for examples of holistic mentorship see [39] and [20] to read more about the science of effective STEM mentorship). This will inform how to support their unique needs and provide robust support that is equitable while setting reasonable expectations and validating their competence and potential for growth [39].

### Rule 8: Engage with their professional development

A simple first step in engaging with the professional development of your FLI students is to share your STEM journey and how you ended up in your current position. Sharing your STEM journey can provide valuable insights into how to navigate a STEM career and/or academia. For many FLI students, hearing about different education journeys can help fill in some of the unknowns about career and/or educational opportunities that they might not otherwise pursue or have access to.

In addition, consider other ways to engage in FLI students’ professional development. For example, inviting them to attend a research conference can give them perspective into what a career in science looks like. You can invite them to engage in research partnerships and coauthor papers or conference proceedings. Students with faculty-mentored undergraduate research experiences are more likely to complete STEM degrees and be accepted into graduate programs [40], as research experience enriches their credentials and application materials.

If you are not a faculty member, you can share materials such as Applying to Graduate School: Tips, Timeline, and Tools of the Trade [41] or consider connecting them with faculty or former students who would be willing to talk to them about careers in STEM. Consider hosting workshops and/or advising sessions on topics like securing fellowships or applying to a summer research experience for undergraduates (REU) programs. Such opportunities provide transparency into the types of resources available to finance their education or research. Although application requirements may be publicly available, the details that make for a competitive fellowship or grant application may elude FLI students. Hosting individual review appointments can help coach FLI students through revisions of their applications and normalize the iterative writing process. These strategies can be influential in connecting FLI students to graduate opportunities and careers in STEM and are essential for their retention and success.

### Rule 9: Connect FLI students to other mentors

Mentorship is extremely important for FLI students as they likely have fewer individuals in their networks who have attended college [42], leaving limited options when they require guidance. It is well established that for underrepresented students in STEM, mentors provide a crucial intervention in demystifying the academic and social complexities of higher education [42–47]. Peer mentorship [46] as well as having multiple mentors can contribute to a strong support system for FLI students navigating STEM.

The FLI identity is often invisible—for employees in mid to late-career stages, it may not feel natural at first to self-disclose about early life experiences and upbringing [48], so it’s important to build spaces to talk about it openly. A key strategy is to ask colleagues, especially faculty members, who identify as former FLI students to connect with your current FLI students [49]. Amplifying the vast network of FLI-identified people at all levels within the institution is crucial to role modeling and providing critical services that set students up for success. It also enriches the FLI network on campus by including student, faculty, and staff community members.

A broad network is an excellent way to ensure FLI students have multiple sources of support when navigating their higher education journeys. For resources on identifying and connecting with mentors, you can refer students to the following tools: Why You Need Multiple Mentors [50] and the Mentor Map Worksheet [51].

### Rule 10: Advocate on behalf of FLI students for institutional support

The rules and strategies listed above highlight ways to offer interpersonal support; yet considerable systemic and institutional support is needed to address structural barriers that impede accessibility in STEM. For example, many institutions lack transparency in providing guidance on degree requirements, academic milestones, support services, seeking mentorship, and how to navigate institutions that are unfamiliar to them. Importantly, when FLI students fail, it is not a failure of the student but a failure of the institution’s ability to adequately address their unique needs.

You can advocate on behalf of FLI students internally as a faculty member, student services professional, or trusted campus leader. The Student Affairs Forum within the Education Administration Programs suggests proactive, campus-wide approaches—leveraging resources and personnel from across the campus community—to ease transitional barriers for FLI students and support institutions in their efforts to improve the experiences and outcomes for FLI students [52]. As you become familiar with the needs and concerns of FLI students, start building a network of support or join existing networks where you can openly share institutional knowledge and strategies. Collaboration with other advocates and stakeholders will contribute to developing system-wide approaches to supporting FLI students and their degree completion. For more examples of how to advocate for systemic changes that support FLI students, see [53,49].

## Conclusion

FLI students represent a diverse and often invisible community, especially in STEM. FLI STEM students have the potential to be a value-add to your research community through their resourcefulness, adaptability, and collaborative nature [3]. Supporting FLI STEM students in the academy has limitless potential for furthering innovative scientific discoveries while solidifying the commitment to diversity, equity, inclusion, and belonging in STEM disciplines [54]. As mentors of students who experience socioeconomic challenges, it is important to hear those challenges and improve your own practices and strategies. We intend for these 10 simple rules to serve as a starting point to explore and inform your journey in supporting FLI STEM undergraduate students which will contribute to the diversification of STEM fields and create inclusive research environments.
